# *Mycobacterium tuberculosis*–Human Immunodeficiency Virus Infection and the Role of T Cells in Protection

**DOI:** 10.3390/vaccines12070730

**Published:** 2024-06-30

**Authors:** Kiana Hosseinian, Amir Gerami, Melody Bral, Vishwanath Venketaraman

**Affiliations:** Department of Basic Medical Sciences, College of Osteopathic Medicine of the Pacific, Western University of Health Sciences, Pomona, CA 91766, USA

**Keywords:** tuberculosis, *Mycobacterium tuberculosis*, HIV, TB, TB-HIV coinfection, immune response, cytokines, IFN-γ, CD4+ T cells, CD8+ T cells, granuloma, vaccine development

## Abstract

Tuberculosis (TB), primarily caused by *Mycobacterium tuberculosis* (*M. tb*), remains a widespread fatal health issue that becomes significantly detrimental when coupled with HIV. This study explores the host’s innate and adaptive immune system response to TB in HIV immunocompromised patients, highlighting the significant role of CD8+ T cells. While the crucial role of macrophages and cytokines, like TNF-α and IFN-γ, in managing the host’s immune response to *M. tb* is examined, the emphasis is on the changes that occur as a result of HIV coinfection. With the progression of HIV infection, the primary source of IFN-γ changes from CD4+ to CD8+ T cells, especially when latent TB advances to an active state. This study sheds light on the necessity of developing new preventative measures such as vaccines and new treatment approaches to TB, especially for immunocompromised patients, who are at a higher risk of life-threatening complications due to TB-HIV coinfection.

## 1. Introduction

Tuberculosis (TB), caused by the bacterium *Mycobacterium tuberculosis* (*M. tb*), remains a significant global issue and is responsible for the death of millions of people annually. In 2022, the World Health Organization (WHO) reported that 1.3 million individuals died while infected with TB, of which 167,000 were coinfected with the human immunodeficiency virus (HIV). As per the WHO, if left untreated, approximately 60% of TB-infected individuals who are HIV-negative and nearly all of those who are HIV-positive will succumb to the disease. It has been recently noted that approximately one-third of global acquired immunodeficiency syndrome (AIDS)-related fatalities are caused by *M. tb*, making it the primary cause of death among those living with HIV. In 2022, there were an estimated 167,000 deaths due to TB-HIV coinfection, and about 13% of all new TB cases are also HIV-coinfected [1]. According to a nationwide retrospective cohort study, 9% of HIV patients receiving treatment also had TB [2]. In 2014, the WHO outlined several strategies in their End TB Strategy, which aims to reduce the global mortality and incidence of TB [1]. In America, both its incidence and mortality rates have gradually risen in recent decades [3]. While approximately 1.8 billion people worldwide are infected with TB, most undergo complete resolution of the disease process, and only a small subset progresses to active TB infection at some point in their lives [4]. Currently, our knowledge about TB’s pathogenesis and infection process is developing, but there are still unclear avenues that need to be discovered. In fact, the existence of both active and latent TB infection has been globally accepted by experts. Interestingly, the interplay between the host defense system and *M. tb* has been of great interest to scientists in recent years.

*M. tb* is known as a facultative intracellular pathogen that resides within macrophages after being introduced into the host body. During the early stages of TB infection, members of innate immunity, most notably the alveolar macrophages and dendritic cells, recognize *M. tb*’s surface markers, known as pathogen-associated molecular patterns (PAMPs), via their own membrane-associated pattern recognition receptors, which include toll-like receptors (TLRs). PAMPs’ chemical compositions range from carbohydrates to lipids, lipoproteins, nucleotides, and proteins. This diversity necessitates a correspondingly diverse set of host receptors that can be found both intracellularly and on the cell surface. Upon interaction between PAMPs and TLRs, intracellular signaling pathways are activated, inducing the production of pro-inflammatory cytokines such as tumor necrosis factor (TNF-α), interleukin-1 (IL-1), IL-12, etc. In fact, the interaction between PAMPs and their receptors is a highly complex process. Multiple interactions occur simultaneously, in which various PAMPs and their corresponding receptors are engaged at the same time [5]. In contrast to other infectious agents that are engulfed by host macrophages during the primary infection, *M. tb* averts the development, differentiation, and fusion of phagosomes into lysosomes. Remarkably, the phagosome that houses the bacteria resembles an early endosome and does not go through acidification or destruction intracellularly. Thus, the infected macrophage can potentially undergo cell necrosis, apoptosis, or, in some instances, survival. It is only in apoptosis that the bacteria are effectively destroyed intracellularly due to an intact cell membrane. Otherwise, the living bacteria are released from the macrophages and have the potential to infect neighboring cells [6]. Once the innate immune system of the host fails to clear the initial phase of TB infection or *M. tb* persists, the adaptive immune system becomes activated by specific T cells within 2–3 weeks. Dendritic cells and natural killer cells play a crucial role as intermediaries between the innate and adaptive immune systems. These cells, along with macrophages, present bacterial antigens to naive T cells upon translocating to regional lymph nodes. Following this antigen presentation, CD4+ T cells are activated, migrate to the lung tissue, and inhibit the growth of *M. tb*.

Among different lineages of T helper cells, Th1 and Th17 are predominantly noteworthy for their key roles in facilitating protection and immunity during TB infection. Various cytokines such as IL-12 and IFN-γ are crucial for the differentiation of naïve T lymphocytes into the Th1 subtypes. These cytokines are mostly secreted from antigen-presenting cells. In particular, the interaction between IL-12 and its receptor leads to the induction of STAT4 and subsequently T-bet, known to be the principal regulator of Th1 cells. IFN-γ, on the other hand, induces STAT1, which, with STAT4, activates T-bet in the Th1 cells synergically. Conversely, IL-4 and IL-10 have been shown to be inhibitors of Th1 cell differentiation [7]. As a result, Th1 cells secrete more IFN-γ, IL-2, and TNF-α. These cytokines play crucial roles in macrophage activation and are responsible for cell-mediated immunity and phagocyte-dependent immune responses [8].

CD4+ T cells exert regulatory control over CD8+ T cells at different stages, such as priming, expansion, migration, effector function, survival, and the formation of memory [9]. CD4+ T cells enhance the translocation of CD8+ T cells to the mucosal tissue’s microenvironment. This induction effect is mediated through IFN-γ chemokines. CD8+ T cells, in turn, are capable of recognizing and responding to essential signals crucial for their function and prolonged residence [10]. The importance of the adaptive immune system, especially the T cells, during TB infection becomes apparent when examining individuals coinfected with HIV. Such patient populations are more susceptible to developing latent TB and experiencing the reactivation of *M. tb* compared to their counterparts who are not infected with HIV. As HIV infection progresses and the number of T cells declines, the susceptibility to TB infection increases. Within the host, the interaction between *M. tb* and HIV results in a decline in host immunological responses. In fact, in high-risk regions, the coinfection of *M. tb* with HIV appears to be the greatest risk factor for the development of active TB. Interestingly, this increased susceptibility is not limited to the primary infection but also applies to reactivation and reinfection in those with a latent TB status. Lastly, infection with *M. tb* adversely influences the host immune response to HIV, hastening the progression from HIV infection to AIDS [11].

## 2. Materials and Methods

Despite current preventative measures against TB, it remains a widespread fatal disease, especially in the immunocompromised. We intend to further explore the host–pathogen interactions, pathogenesis, and current vaccine regimens to bridge the knowledge gap on the two disease processes and to guide efficacious vaccine development. Any challenges that arose were initially resolved through group discussions, culminating in a final decision by Dr. Venketaraman to ensure consistency and fluency throughout the paper. These articles were chosen according to the relevance of the topic and the credibility of the source. Although the majority of this paper is from past research and literature, we tried to introduce novel concepts, such as the incorporation of current vaccine trials for TB for the immunocompromised, as well as the factors to consider when developing these vaccines. The keywords that were used to obtain these articles were as follows: tuberculosis, *Mycobacterium tuberculosis*, HIV, TB, TB-HIV coinfection, immune response, cytokines, IFN-γ, CD4+ T cells, CD8+ T cells, granuloma, and vaccine development. Resources included random control, case–control studies, literature reviews, meta-analysis, and studies conducted on individuals with known TB, immunocompromised individuals, or those who were both. Different populations were included, regardless of age group, ethnicity, socioeconomic status, or geographic location. Articles were excluded due to their non-relevance, not having been peer-reviewed, or their use of a non-English language. Articles specific to vaccination were excluded if they had a publication date before 2019. The literature involved in this review was mainly acquired by the use of the following databases: PubMed, CDC, WHO, and Google Scholar. 

## 3. Results

### 3.1. M. tb’s Entry and Initial Response

*M. tb* is primarily transmitted through respiratory and aerosolized droplets. The process begins with the inhalation of respiratory droplets, which triggers innate immune cells, notably alveolar macrophages, to act as the first line of defense by phagocytosing these droplets. PAMPs on *M. tb* are recognized via a variety of receptors to mediate opsonic and nonopsonic bacterial uptake. *M. tb* expresses a variety of known or putative TLR ligands, and TLR-2, TLR-4, and TLR-9 have been implicated in host recognition of *M. tb* [12]. Subsequently, these macrophages release TNF-α, initiating the formation of granulomas, which represent the earliest defense mechanism against this pathogen. Various innate cytokines, including CCL2, IL-6, and TNF-α, are increased during the initiation of mycobacterial infection. These cytokines play a role in granuloma formation, pathophysiology, and local immunity in the lungs during *M. tb* infection [13].

Studies have shown that reactivation of pulmonary TB occurs in latently infected mice upon the neutralization of TNF-α [14]. This demonstrates that TNF-α is required throughout the life of the infected host. Upon neutralization, less defined granuloma formation is seen, along with increased bacterial infection in the lungs and extrapulmonary sites such as the liver and spleen. Enhanced histopathology is also observed in TNF-α-neutralized mice, supporting the importance of regulated cellular interaction during *M. tb* infection [15]. A growing issue revolves around the connection between the utilization of TNF-α inhibitors and a heightened likelihood of *M. tb* reactivation. It was observed that treatment with TNF-α inhibitors resulted in the progression of *M. tb* from latent to reactivation. This explains why healthcare providers undergo extensive screening for *M. tb* prior to initiating therapy in those individuals with autoimmune diseases who are subjected to anti-TNF-α therapy [16].

**Figure 1:**
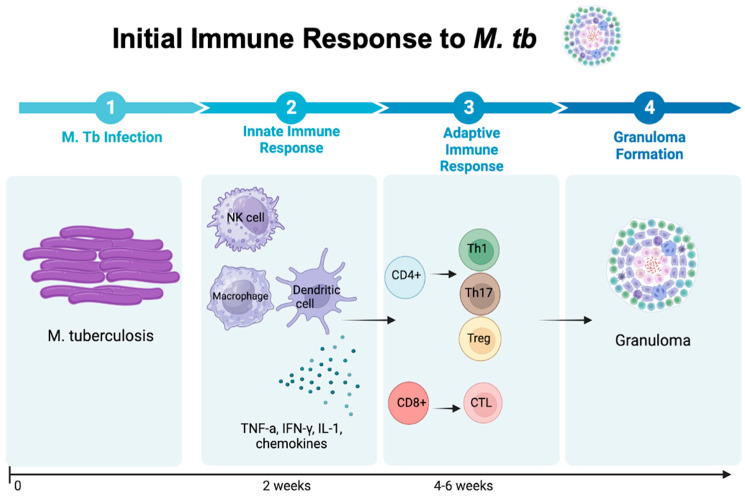
The introduction of *M. tb* into the host cell initiates the innate immune response, involving a coordinated effort of macrophages, natural killer cells, and dendritic cells. These immune cells release pivotal cytokines such as TNF-α, IFN-γ, IL-1, and other chemokines, subsequently activating CD4+ and CD8+ T cells to release their specific cytokines. This orchestrated immune cascade results in the formation of granulomas, representing the hallmark aspect of the host’s strategy to confine and regulate the bacterial presence [20].

Granulomas consist of monocytes, macrophages, epithelioid cells, and multinucleated giant cells and have the role of containing bacterial spread beyond the lungs while also establishing a site for long-term bacterial persistence. The granuloma response induces necrosis, playing a pivotal role in the survival and spread of bacteria, therefore contributing to the disease’s severity and morbidity [17]. Particularly in TB granulomas, the mature macrophages can undergo a distinct transformation into epithelioid cells (also known as epithelioid histiocytes). TB granulomas are distinguished from granulomas of other diseases through their characteristic regions of necrosis, known as caseum. In addition to macrophages, many other cell types also populate the granuloma, such as neutrophils, dendritic cells, B and T cells, natural killer cells, fibroblasts, and cells that secrete extracellular matrix components [18]. Within the granuloma, *M. tb* can remain dormant for several decades, leading to latent infection, and is present in about 90% of infected individuals. Chemokines play a pivotal role in attracting neutrophils, monocytes, and lymphocytes to the granuloma site. The Th1 subset of CD4+ T cells releases IL-2, triggering T-cell proliferation. These activated T cells release IFN-γ, which in turn transforms monocytes into inflammatory macrophages, which are essential for controlling *M. tb.* This process is illustrated in Figure 1. 

For individuals with HIV who experience CD4+ T-cell depletion and thus alterations in their immune response, the formation of granulomas is of particular interest. A systematic review and meta-analysis carried out by Diedrich et al. summarized findings on how HIV-1 changes *M. tb* granuloma formation. They reported more poorly formed granulomas in those with lower peripheral CD4+ counts. It is worth noting that HIV-1 has not been shown to change the presence of caseous granulomas. The analysis also concluded that CD3+, CD4+, or CD8+ T cells along with the macrophage counts within granulomas were highly variable. Overall, HIV-1 increased the *M. tb* count in granulomas [19].

IFN-γ plays a crucial role in promoting apoptosis of cells infected with *M. tb*. IFN-γ is produced mainly by the CD4+ Th1 subset upon encountering *M. tb* antigens. This cytokine supports and strengthens T-cell responses, particularly those aimed at fighting infection. IFN-γ mediates the production of reactive oxygen and nitrogen intermediates, blocks antigen presentation by downregulating the expression of major histocompatibility complex molecules, induces dendritic cell migration, and recruits Th1 cells to the lungs by modulating chemokine production [21]. Macrophages activated with IFN-γ induce greater inflammasomes. *M. tb* mutations in the IFN-γ gene or variations in IFN-γ receptor expression are often associated with immune deficiencies and an increased susceptibility to *M. tb* infection. Interferon-gamma release assays (IGRAs) represent blood tests performed outside the body to assess the immune response of T cells, specifically testing their release of IFN-γ. Within numerous countries, two primary commercial IGRAs exist: the QuantiFERON-TB Gold In-Tube assay and T-SPOT. A positive indication of *M. tb* infection in an individual is determined when the IFN-γ reaction to TB antigens surpasses the designated test threshold, accounting for the background IFN-γ response measured in a negative control.

### 3.2. HIV’s Entry and Systemic Immunosuppression

In the context of immunodeficient susceptibility in patients with TB, coinfection with HIV has been identified as a significant prognostic factor. HIV, a retrovirus with a single-stranded RNA genome belonging to the Lentivirus family, triggers substantial inflammation in the body and is mainly transmitted sexually or through sharing needles. HIV primarily targets CD4+ T cells, compromising the body’s adaptive immune defense system. The initial symptoms of HIV infection can range from flu-like symptoms, dementia, and depression to ulcers, neuropathy, and encephalitis. Interestingly, it increases susceptibility to other fatal infections such as pulmonary TB, mainly by weakening the adaptive immune response.

At the initial stage of entry, viral particles bind to the target cell, CD4+ T cells, either through the viral envelope or other cell membrane attachment factors [22]. This interaction exhibits varying specificity, involving the binding of specific receptors such as CCR5, CXCR4, and α4β7 integrin to the viral envelope or nonspecific attachment to cell surface heparan sulfate proteoglycans. Viral envelope proteins, gp120 and gp41, bind to CD4+ T cells, inducing envelope changes that enable the fusion peptide of gp41 to attach to the target membrane of CD4+ T cells. Moreover, different HIV strains bind to the specific coreceptor CXCR4 on CD4+ T cells. Upon the completion of membrane fusion, the viral genome and proteins can be transported inside the cell. After viral entry, HIV initiates replication within CD4+ T cells, leading to a gradual decline in the T-cell subpopulation. This process is facilitated either through direct attack or secondary immune activation and inflammation [23]. Given that HIV accelerates both the growth and destruction of T cells, there is a continuous replenishment of T cells from the thymus. However, as the viral load escalates to billions, an expanding pool of infected cells exacerbates the depletion of other subtypes, consequently heightening the turnover of T cells [24]. Moreover, it has been observed that activated T cells have a remarkably short lifespan, quickly lost to apoptosis or activation-induced death, further contributing to turnover.

In other words, persistent host immune activation and subsequent inflammation during HIV infection have severe negative effects on the host immune system and patient outcomes. This imbalance alters leukocyte activity and cytokine levels, contributing to disease progression. As mentioned earlier, HIV’s preference for targeting and killing activated CD4+ T-helper cells results in altered cell populations and disrupts T-cell balance, impairing the host’s ability to defend against various pathogens, including *M. tb*. Elevated CD4+ T-cell activation, coupled with a high HIV load, accelerates cell death and further infection. Additionally, CD4+ T-cell depletion triggers an immune system response, stimulating the activation and proliferation of surviving cells, which become new targets for the virus [25].

### 3.3. The Role of CD8+, Treg, and Th17 Cells in M. tb’s Immune Defense

Previous studies have emphasized the association between active TB infection and the facilitation of HIV infection and its progression to AIDS. This phenomenon can be related to their shared anatomical reservoirs, such as the lungs. Researchers have investigated the different modalities involved in this process. As previously mentioned, active TB infection accelerates the loss of CD4+ T cells, increasing the body’s susceptibility to opportunistic infections such as HIV [26,27]. The inflammatory immune response against *M. tb* boosts HIV’s replication in the blood and within specific host immune cells, such as lymphocytes and macrophages. Various in vitro studies have shown that *M. tb* upregulates HIV replication through cytokine-mediated pathways [28,29,30,31]. Furthermore, HIV transcription is affected by the pro-inflammatory chemokines and cytokines released in response to *M. tb* infection. More interestingly, the terminal differentiation of CD4+ T cells at TB disease sites leads to the expression of CCR5, making these cells more susceptible to HIV infection [32].

CD4+ and CD8+ T cells are the primary sources of IFN-γ production within the host body, with CD4+ T cells being the major contributors. Interestingly, although both cell types play a part in enhancing the host immunological defense against intracellular bacterial infection, the complete absence of either cell type does not significantly compromise the IFN-γ production of the other cell type. In fact, in the absence of CD4+ T cells, activated CD8+ T cells exhibit the potential to provide immune protection against secondary mycobacterial infections, such as reinfection or reactivation of the latent TB. Hence, the IFN-γ production, response, and activation of CD8+ T cells can occur independently of CD4+ T cells [33]. 

In the host body of individuals coinfected with HIV, the predominant source of IFN-γ production may shift to CD8+ T cells depending on the CD4+ T-cell count in the body. In advanced disease stages, CD8+ T cells may even become the primary and/or the only source of IFN-γ production, providing immunological protection against secondary infections of *M. tb.* However, it is crucial to note that CD4+ T cells also play a crucial role in promoting the activity and survival of effector CD8+ T cells during the primary responses. This function is particularly critical in maintaining effective CD8+ T-cell responses during chronic infections, which may become compromised in those with advanced HIV infection. Therefore, the significance of CD8+ T cells in the response to TB infection becomes evident in the context of TB-HIV coinfection. Regardless of HIV infection status, the activation of CD8+ T cells specific to *M. tb* antigens would enhance immunological protective responses, especially in those with a compromised CD4+ T-cell count.

**Figure 2:**
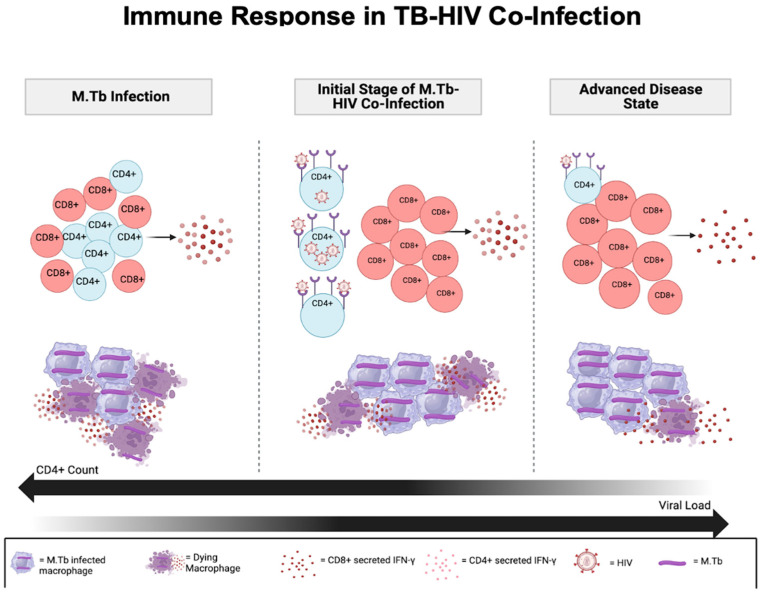
Both CD4+ and CD8+ T cells contribute to IFN-γ production in the body, with CD4+ T cells being the primary contributors. IFN-γ and other cytokines play a crucial role in macrophage activation and are responsible for cell-mediated immunity and phagocyte-dependent immune-protective responses in *M. tb* infection. In the context of coinfection with HIV, the number of CD8+ T cells increases, while CD4+ T count declines as the disease progresses. In the advanced disease state, CD8+ T cells become the major source of IFN-γ production, which is crucial for combating TB infection [41].

In a study conducted by Van Pinxteren et al., it was revealed that in a mouse model, the CD4+ subset exhibited remarkable activity during the acute phase of infection, with limited engagement of the CD8+ cells. Conversely, the CD8+ subset emerged as the primary active cell type among the two subsets during the latent phase of infection. This conclusion was further supported by in vivo experiments involving the depletion of T-cell subsets. Specifically, administering anti-CD4+ treatment during the acute phase resulted in a 6–7-fold increase in the bacterial load in the lungs, whereas anti-CD8+ treatment showed no effect. Similarly, during the latent phase, anti-CD8+ and anti-IFN treatment led to a 10-fold increase in the bacterial load in the lungs, while anti-CD4+ treatment did not significantly alter the bacterial count [34]. This increase in the CD8+ T-cell responses and the association with the *M. tb* load have been established in both human and animal models [35,36,37,38]. Following antiretroviral therapy and therefore CD4+ T-cell restoration, Day et al. observed heightened CD8+ T-cell reactivity to *M. tb* antigens in HIV-positive individuals with depleted CD4+ counts and latent TB infection, as well as in those with active TB [39]. The enhanced CD8+ T-cell responses observed in HIV-infected individuals could serve as an indication that there is a lack of regulation in the absence of CD4+ T cells. This could potentially be disadvantageous for HIV-infected hosts in combating TB, especially when compared to uninfected individuals [40]. This process is depicted in Figure 2. 

Another dynamic aspect of the host defense system worth mentioning is the involvement of the balance between the Treg and Th17 subtypes of T cells. Th17 cells are primarily known for the production of IL-17, which stimulates the production of other pro-inflammatory cytokines and chemokines, aiding the body in mounting a protective yet pro-inflammatory immune response against pathogens. On the other hand, Tregs have been associated with various inflammatory and autoimmune processes, as well as some infectious ones. Key cytokines contributing to the immunosuppressive function of Tregs include IL-10, TGF-β, and IL-35. Tregs interfere with T-cell activation by the dendritic cells through a CTLA-4 dependent mechanism. Maintaining balance with the Th17 cells is widely regarded as pivotal in these conditions. In other words, Tregs and Th17 cells are closely interconnected. In chronic infectious diseases such as TB, a delicate balance between pro- and anti-inflammatory responses exists. While Th1 and Th17 cells are essential for controlling *M. tb* infection, the inflammatory cascade can eventually become detrimental to the host. The establishment of this balance between opposing forces could potentially determine the extent of the damage inflicted on the lung tissue or distant organs during the advanced stages of the disease. Coinfection with HIV could further exacerbate these processes, delaying the establishment of equilibrium between the two sides and exacerbating tissue destruction within the microenvironment [42]. 

Moreover, among the cytokines that affect CD4+ and CD8+ T-cell activities in HIV infection, IL-10 plays a direct inhibitory role. Regarding CD8+ T cells, IL-10 has been found to decrease their antigen sensitivity during chronic viral infections through specific signaling pathways. Consequently, the CD8+ T cells’ function against viral infection is reduced due to their increased activation threshold by IL-10. Thus, inhibition of the IL-10 regulatory pathway can be considered one of the treatment approaches to chronic viral infections such as HIV [43]. The engineering of novel vaccines designed to promote CD8+ T-cell responses is also critical for protecting individuals at the highest risk of TB, particularly those coinfected with HIV [44]. *M. tb*’s significant role in HIV infection’s disease progress emphasizes the necessity for new treatment approaches, especially vaccinations, that can decelerate the disease progression to AIDS and decrease the mortality rate in TB-HIV-coinfected patients [45].

The Bacillus Calmette–Guérin (BCG) vaccine has been a commonly used live vaccine for TB since it was developed in 1921 [46]. However, there are limitations and drawbacks associated with the BCG vaccine. It is contraindicated in immunocompromised patients due to it being a live vaccine. According to the WHO, BCG-specific T-cell responses are defective in HIV-infected individuals, and this leads to little or no immunity against TB in these immunocompromised patients [47]. However, new research has shown that recombinant BCG (rBCG) and different routes of administration, despite the traditional BCG vaccine being intradermal, can be more effective against TB while being safer for the immunocompromised.

rBCG vaccines, such as VPM1002 (also known as rBCG ΔureC::hly), are genetically modified versions of the traditional BCG vaccine. The VPM1002 vaccine’s genetic modification includes the deletion of urease C (ureC) and the insertion of listeriolysin O (LLO), a protein derived from Listeria monocytogenes. These genetic modifications make VPM1002 a beneficial vaccine for comorbid TB-HIV through enhancing antigen presentation and CD8+ and AIM2 inflammasome activation using LLO and inducing further IFN-γ production, which is especially beneficial for the immunocompromised [48]. This VPM1002 vaccine has shown to be promising for immunocompromised populations due to being attenuated, besides the genetic modifications mentioned above.

According to a clinical trial in South Africa, VPM1002 is safe for newborn infants, regardless of their HIV exposure history, and has a lower risk of side effects compared to BCG [49]. Phase II/III trials are ongoing to further evaluate the VPM1002 vaccine’s efficacy in preventing TB, particularly for the immunocompromised [50]. There are preclinical studies on variations of VPM1002, such as ΔnuoG, that have demonstrated further protection against TB by increasing apoptosis of infected cells and reducing bacterial loads. However, further clinical trials are required to assess the efficacy and safety of this variation. 

Different routes of administration for BCG, instead of the traditional intradermal route, have also shown to be successful in improving its efficacy and safety in high-risk populations. Preclinical studies on the intranasal route of administration for BCG have shown the potential to provide improved immunization through targeting mucosal immunity and consequently inducing local immunity in the respiratory tract, which is the primary site of TB infection. This route of administration is especially beneficial for the immunocompromised since it mainly induces local immunity and has a lower risk of the side effects associated with systemic dissemination [51]. Researchers are currently working on several clinical trials to further evaluate intranasal BCG in larger populations and methods to facilitate its widespread use [52].

Another safe vaccination option against TB for HIV-infected patients may be protein subunit or inactivated mycobacterial vaccines. M72/AS01E is a subunit candidate vaccine that could be a potentially groundbreaking innovation, especially for HIV individuals [53]. The M72/AS01E candidate vaccine is a fusion protein, constructed from two *M. tb* immunogenic antigens Mtb39A and Mtb32A, combined with the adjuvant system AS01E. The AS01E adjuvant enhances the immunogenicity of the M72 antigens, leading to increased activation of the CD8+ T cells and the production of cytokines such as IFN-γ, both essential immune system components for protection against TB [54,55]. Furthermore, according to a meta-analysis of five randomized clinical trials, the M72/AS01E subunit vaccine had an abundance of polyfunctional M72-specific CD4+ T cells in the vaccine group versus the control group. This synergistic M72/AS01E-induced CD8+ and CD4+ T-cell response induction makes M72/AS01E a promising vaccine [56]. Currently, phase 3 trials for this vaccine are progressing. These trials, aiming to assess the efficacy and safety of the vaccine across diverse populations, include up to 20,000 participants from various populations, such as those who are HIV immunocompromised, and multiple countries, such as South Africa, Zambia, Malawi, Mozambique, Kenya, Indonesia, and Vietnam.

In 2017, the vaccine components from the APPROACH study were utilized in the Imbokodo trial [57,58] in Southern Africa, which involved 2637 participants in a phase IIb clinical trial. However, the Imbokodo study was terminated prematurely due to its disappointing efficacy. Despite this setback, there is optimism surrounding the Mosaico trial, initiated in 2019, which evaluates the effects of Ad26.Mos.HIV and an adjuvanted clade C gp140 protein vaccination in North America, Latin America, and Europe. Although there have been many attempts to develop an effective vaccine, Mosaico and the HVTN 702 trials are the only two HIV vaccine efficacy trials in the past decade that enrolled over 100 participants and progressed to phase III [59].

Furthermore, determining the effectiveness of vaccines targeting CD4+ T cells versus those targeting CD8+ T cells is another question to explore in designing TB vaccines. Although CD4+ T-cell induction is a common approach in TB vaccines, some studies have failed to prove that polyfunctional CD4+ T cells are sufficient to protect against *M. tb* infection. Various functional characteristics of T cells, such as IL-17 production, and other major cells, such as classically restricted CD8+ T cells, might be the underlying reason for the correlation of CD4+ T cells with immunity to *M. tb* in humans [60]. Since CD8+ T cells play a significant role in fighting TB, especially in the immunocompromised population, a vaccine that can dominantly induce a CD8+ T-cell response is worthwhile and preferred above BCG, which mildly affects these T cells. However, these studies have been limited because few MHC class I mycobacterial antigens have been identified [61]. Nyendak et al. report that “some of the vaccine-elicited CD8+ T cells failed to recognize epitopes displayed by the *M. tb*-infected cells due to limited access to the HLA-Ia processing machinery during the course of *M. tb* infection in vivo” [62]. Finally, research demonstrates that a synergistic combination of CD4+ and CD8+ T-cell-inducing vaccines has been the most successful for preventing TB.

## 4. Conclusions

TB, caused by *M. tb*, remains a significant global health concern, resulting in millions of deaths annually. Although our current understanding of the disease process of TB is advancing, there remain unexplored aspects that require further study. *M. tb*’s pathogenesis begins with invasion into the lungs and the evasion of the host immune response, triggering granuloma formation, leading to a complex interplay between the pathogen and the host’s defense mechanisms during TB infection. It is important to appreciate the pathogenesis of *M. tb* when examining individuals coinfected with HIV. HIV patients are considered immunocompromised and therefore are more susceptible to latent TB infection. 

This depletion in the host immune response accelerates the progression from HIV to AIDS. IFN-γ is produced by the CD4+ and CD8+ T cells and plays a vital role in macrophage activation. It has been studied that those with *M. tb* and HIV coinfection have increasing numbers of CD8+ T cells and decreasing numbers of CD4+ cells. In the later stages of the disease, CD8+ T cells emerge as the primary suppliers of IFN-γ, playing a pivotal role in protecting against *M. tb* infection. Understanding these processes is crucial for developing effective strategies for TB prevention and treatment. This paper emphasizes the important role of CD8+ T cells in fighting against pathogens, specifically in individuals coinfected with TB and HIV, and suggests prophylactic vaccinations should be aimed at increasing the number of CD8+ T cells. Because of HIV’s mechanism of depleting T cells, it may be worthwhile to develop protein subunit or inactivated mycobacterial vaccines that could provide a safer vaccination choice for HIV-infected individuals against TB. Understanding the immune responses against *M. tb* and the impact of HIV coinfection highlights the urgency for further research into targeted immunotherapies and vaccines. Such advancements are necessary for battling the global crisis of *M. tb* and improving the outcomes for high-risk individuals, paving the way for more effective prevention and treatment strategies in the future.

## Data Availability

No new data were created or analyzed in this study. Data sharing is not applicable to this article.
